# Spinach-associated *Escherichia coli* O157:H7 Outbreak, Utah and New Mexico, 2006

**DOI:** 10.3201/eid1410.071341

**Published:** 2008-10

**Authors:** Juliana Grant, Aaron M. Wendelboe, Arthur Wendel, Barbara Jepson, Paul Torres, Chad Smelser, Robert T. Rolfs

**Affiliations:** Centers for Disease Control and Prevention, Atlanta, Georgia, USA (J. Grant, A.M. Wendelboe, A. Wendel); Wisconsin Department of Health and Human Services, Madison, Wisconsin, USA (A. Wendel); New Mexico Department of Health, Santa Fe, New Mexico, USA (A.M. Wendelboe, P. Torres, C. Smelser); Utah Department of Health, Salt Lake City, Utah, USA (J. Grant, B. Jepson, R.T. Rolfs); 1 Current affiliation: Agency for Toxic Substances and Disease Registry, Atlanta, Georgia, USA.; 2 Current affiliation: University of Oklahoma Health Sciences Center, Oklahoma City, Oklahoma, USA.

**Keywords:** Escherichia coli, Spinach oleracea, disease outbreaks, Escherichia coli O157, case-control studies, electrophoresis, gel, pulsed-field, Utah, New Mexico, Wisconsin, dispatch

## Abstract

In 2006, Utah and New Mexico health departments investigated a multistate cluster of *Escherichia coli* O157:H7. A case–control study of 22 case-patients found that consuming bagged spinach was significantly associated with illness (p<0.01). The outbreak strain was isolated from 3 bags of 1 brand of spinach. Nationally, 205 persons were ill with the outbreak strain.

On September 13, 2006, health officials from several states independently notified the Centers for Disease Control and Prevention (CDC) about clusters of *Escherichia coli* O157:H7 infections and a suspected association with spinach. *E. coli* O157:H7 expresses 1 of 2 types of Shiga toxin and can cause severe gastrointestinal infections and hemolytic uremic syndrome (HUS).

A multistate outbreak investigation, involving 26 states, was initiated on September 14. The US Food and Drug Administration (FDA) and CDC advised consumers not to eat bagged spinach (*1*,*2*). The Utah (UDOH) and New Mexico Departments of Health conducted a case-control study to characterize the outbreak and a laboratory investigation to test spinach eaten by case-patients for contamination. This report focuses on the investigation conducted in the 2 states.

The case definition for a laboratory-confirmed illness was culture-confirmed *E. coli* O157:H7 infection in a Utah or New Mexico resident with illness onset during August 1, 2006–October 1, 2006, and a pattern of Xba EXHX01.0124 shown by pulsed-field gel electrophoresis (PFGE). This urgent outbreak investigation did not require institutional review board approval.

## The Study

The UDOH Enteric Disease Case Report Form and a supplemental CDC questionnaire on spinach consumption were administered by local or state public health officials to all participants by telephone. Information collected included date of disease onset, symptoms, treatment, community-based exposures, and a food-item history. Questions referred to the 8–10 days before case-patient symptom onset. Case-patients were first interviewed 3–23 days after illness onset (mean = 11.6 days); follow-up interviews for the questionnaire were completed within 23 days of illness onset.

Two controls per case-patient were matched by sex and age group to prevent confounding from potential differences in diet (*3*); age groups were <4 years, 5–12 years, 13–18 years, 19–64 years, and >65 years. Controls were selected by using sequential-digit telephone dialing based on the matched case-patient’s telephone number. Controls reported no gastrointestinal illness 3 days before and after symptom onset date of their matched case-patient.

Exact matched odds ratios (mOR) and confidence intervals (CI) were calculated by using conditional logistic regression in SAS 9.1 (SAS Institute, Cary, NC, USA). An α of 0.05 was used. No statistical analyses were performed for the categories “spinach brand” or “location spinach was eaten” because of insufficient data and inability to generate point estimates; we provide only descriptive evaluations of these variables. Only persons who indicated definite exposure to a single brand of spinach were included in the evaluation of brand.

The Utah Public Health Laboratories (UPHL) and New Mexico Scientific Laboratory Division (NMSLD) provided analytic testing services for all clinical and spinach samples. Public health officials collected spinach samples in their original packaging from confirmed case-patients. One spinach sample in New Mexico was frozen; all other samples were refrigerated. Modified FDA Bacterial and Analytical Manual methods were used to recover *E. coli* O157:H7 from both clinical and spinach samples (*4*). UPHL used MacConkey broth and NMSLD used Food Emergency Response Network (FERN) broth for enrichment instead of Enterohemorrhagic *E. coli* Enrichment Broth; both laboratories used an additional selective media (CHROMagar, CHROMagar, Paris, France).

The presence of O157 and H7 antigens in clinical and spinach samples was confirmed with latex agglutination typing. UPHL used ProLexLatex Agglutination System (Pro-Lab Diagnostics, Austin, TX, USA); NMSLD used Oxoid Dryspot *E. coli* O157 test kit (Oxoid, Cambridge, UK) and DIFCO H7 antiserum (DIFCO Laboratories, Detroit, MI, USA). All clinical isolates were tested for Shiga toxin genes (*stx1* and *stx2*) by PCR. Clinical and spinach isolates were tested for Shiga toxin expression with the Premier EHEC enzymatic immunoassay Shiga-toxin test kit (Meridian Diagnostics, Inc., Cincinnati, OH, USA). PFGE was performed on *E. coli* O157:H7 cultures from clinical and spinach samples by using standard CDC PulseNet operating procedures (*5*). PCR testing reagents and protocols were provided through the Laboratory Response Network (LRN) and FERN.

Eighteen cases were confirmed in Utah and 5 in New Mexico. Onset dates ranged from August 22 to September 11 (Figure). Shiga toxin 2 was detected in stool samples from all patients. Demographic, clinical, and food exposure information was available for all patients (Tables 1, 2). Fifty-seven percent of case-patients were hospitalized, and 29% experienced HUS (age range 2–60 years). No deaths were reported.

**Figure:**
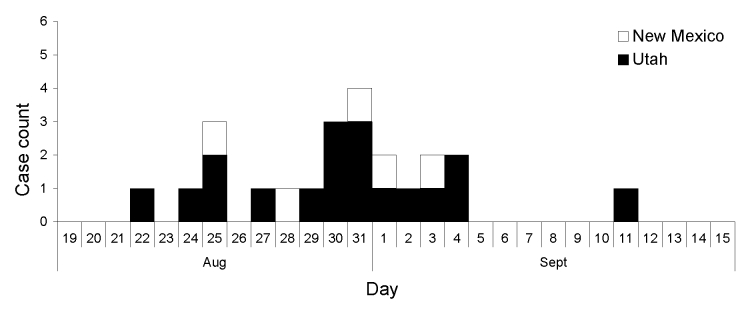
Epidemic curve by date of onset of confirmed cases*, Escherichia coli* O157:H7 spinach-associated outbreak, Utah and New Mexico, 2006.

**Table 1:** Demographic and clinical information on confirmed cases, *Escherichia coli* O157:H7 spinach-associated outbreak, Utah and New Mexico, 2006

Characteristic	No. (%), n = 23
Sex	
M	5 (22)
F	18 (78)
Age group, y	
<4	4 (17)
5–12	5 (22)
13–18	1 (4)
19–64	12 (52)
>65	1 (4)
State of residence	
New Mexico	5 (22)
Utah	18 (78)
Clinical outcome	
Bloody diarrhea	22 (96)
Hospitalized	13 (57)
Hemolytic uremic syndrome	7 (29)

**Table 2:** Bivariate analysis of statistically significant spinach-related exposures during 8–10 d before onset, *Escherichia coli* O157:H7 spinach-associated outbreak, Utah and New Mexico, 2006*

Variable	Case-patients	Controls	Matched OR	95% CI	p value
Ate any spinach					
Yes	18	15	16.5	2.4–710.2	0.0005
No	3	28	1.0		
Ate bagged spinach					
Yes	17	8	18.7	2.8–797.1	0.0001
Other type of spinach	1	2	10.1	0.1–988.8	0.41
No spinach	3	28	1.0		
No. of times ate spinach					
>2	8	2	†	3.6–∞	0.0004
1–2	7	9	7.4	0.8–354.7	0.08
0	3	28	1.0		
Rewashed spinach					
Yes	5	4	1.0	0.08–13.8	1.0
No	9	6	1.0		
Location where spinach eaten‡					
Restaurant	0	6			
Private home	16	10			
Brand					
Ate only brand A	7	1			
Ate only brand B	0	2			

*OR, odds ratio; CI, confidence interval. †Unable to calculate a maximum likelihood estimate for a matched OR. ‡Certain persons reported having eaten spinach both at home and in restaurants.

Matched analyses were performed on 22 case-patients and 44 matched controls. Consumption of bagged spinach (mOR = 18.7, 95% CI = 2.8–797.1, p<0.01) was significantly associated with case status (Table 2). Food items previously implicated as sources of pathogenic *E. coli* were not significantly associated with case status.

No patients, versus 6 controls, reported only eating spinach at a restaurant; 16 patients and 10 controls reported only eating spinach in a private home (Table 2). Washing spinach before eating did not significantly change the odds of being a case-patient (Table 2). No study participants reported eating only cooked spinach; therefore, cooking was not analyzed. Ten case-patients and 3 controls reported definite exposure to single brands of spinach; only brands A and B were reportedly consumed by these patients. Seven case-patients and 1 control reported definite exposure to brand A; 0 case-patients and 2 controls reported definite exposure to brand B (Table 2).

Seven bags of spinach (5 from Utah, 2 from New Mexico) were provided by 7 case-patients. Five bags had been opened and their contents partially consumed. *E. coli* O157:H7, matching the national outbreak strain, was detected by PCR and culture in 2 Utah spinach bags and the New Mexico unfrozen bag. All 3 were open bags of brand A baby spinach. Lot codes were available on 2 bags; both were packed on August 15, 2006, at the same plant, on the same shift, but on different machines.

The 4 bags from which *E. coli* O157:H7 was not detected were also brand A baby spinach and were packed on August 15, 22, 23, and 28, 2006. Two bags that tested negative were eaten by case-patients who reported eating from multiple bags before illness onset.

## Conclusions

Consumption of fresh brand A spinach was associated with *E. coli* O157:H7 infection with both epidemiologic and laboratory data. Washing spinach before consumption did not affect odds of being a case-patient. Possible reasons for this include 1) *E. coli* could be internalized into the plant structure by entering through the roots (*6*), and 2) bacteria are more likely to adhere to cut surfaces of leafy greens (e.g., prepackaged spinach) (*7*). That no case-patients reported only eating spinach in a restaurant suggests that the source of the contaminated spinach did not supply commercial establishments.

The percentage of case-patients in whom HUS developed (29%) was high when compared with that in prior *E. coli* O157:H7 outbreaks (15%–20%) (*8*,*9*). This finding is consistent with studies that associate Shiga toxin 2–expressing *E. coli* with a higher incidence of HUS (*10*,*11*).

Our study was subject to certain limitations. One is potential recall bias since controls had a longer time lag between consumption and interview and less motivation to accurately recall what foods they had eaten. However, overestimation of the association between spinach consumption and illness is unlikely because more controls reported having eaten spinach than were previously identified in surveys of the general population (*12*). Analysis of brand was limited because of poor recall among all participants. The sample size was small, resulting in imprecise effect estimates and, in certain cases, an inability to calculate a measure of association. Exact ORs were used to partially counteract this limitation.

This investigation was conducted in response to a national outbreak of *E. coli* O157:H7 infections with matching PFGE patterns among 205 persons in 26 states (*13*). Less than 2 weeks after its initiation, this investigation provided laboratory and epidemiologic evidence implicating spinach. The FDA used these data to focus its field investigation and interventions and linked the contaminated spinach to samples taken from a stream, cattle manure, and feces from wild pigs on ranches in Salinas Valley, California (*13*). In August 2006, FDA launched a lettuce safety initiative to address recurring outbreaks of *E. coli* O157 infections (*14*). After this outbreak, the initiative was expanded to include all leafy greens.
